# Supplementary material for: Antifungal Activity of Electrochemically Etched Nanotextured Stainless Steel against Candida albicans and Fusarium oxysporum Fungal Cells
Source: ACS Omega. 2025 May 7;10(19):19326–34. doi: 10.1021/acsomega.4c09511 (PMC12096193; doi:10.1021/acsomega.4c09511)
Supplement: Supplementary file 1 [file ao4c09511_si_001.pdf]

Antifungal Activity of Electrochemically Etched Nanotextured Stainless Steel Against *Candida albicans* and *Fusarium oxysporum* Fungal Cells

Anuja Tripathi, Cheick Dosso^, Julie A. Champion*

School of Chemical and Biomolecular engineering, Georgia Institute of Technology, 950 Atlantic Drive, Atlanta, Georgia, 30332, USA

^current address: Department of Chemical Engineering, Carnegie Mellon University, Doherty Hall, 5000 Forbes Avenue, Pittsburgh, PA 15213, USA

*corresponding author: e-mail: julie.champion@chbe.gatech.edu

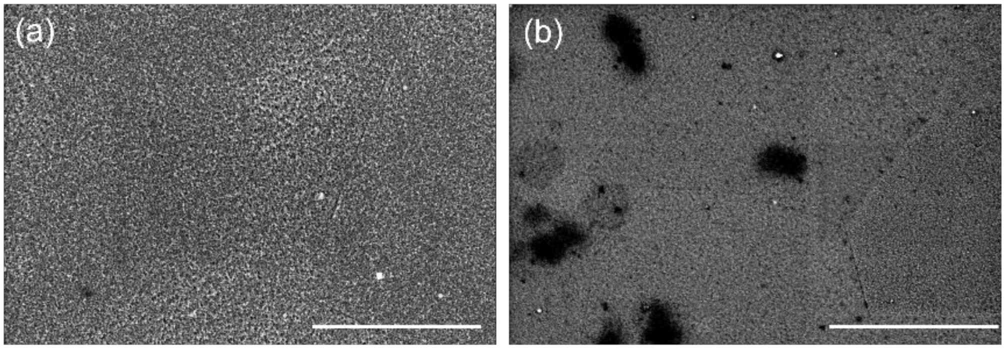

Figure S1: Mechanical tests placing a 640 g static weight on nSS samples for 15 minutes (b). Nanotexture is still apparent, as compared to control nSS samples (a). Debris transferred from static weight was not fully removed by washing and caused charging (black spots). Scale bar is 2 μm.

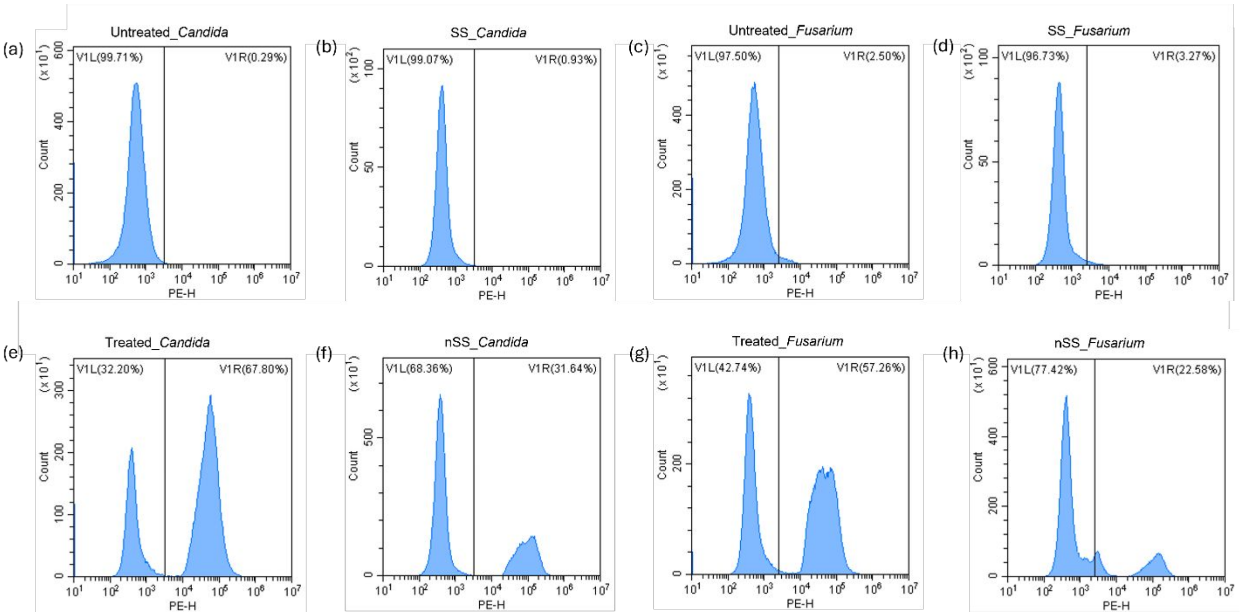

Figure S2: Representative flow cytometry histograms of cells labeled with propidium iodide after incubation with SS and nSS. Gates was drawn from negative control. Cells exposed to nSS exhibited a greater fluorescence shift compared to control samples and those incubated with SS (b, d). Fungal cell controls were left untreated (a, c) and treated with peroxide (e, g). The nSS-incubated cells (f, h) displayed a shift to right, indicating increased dead cell fluorescence.

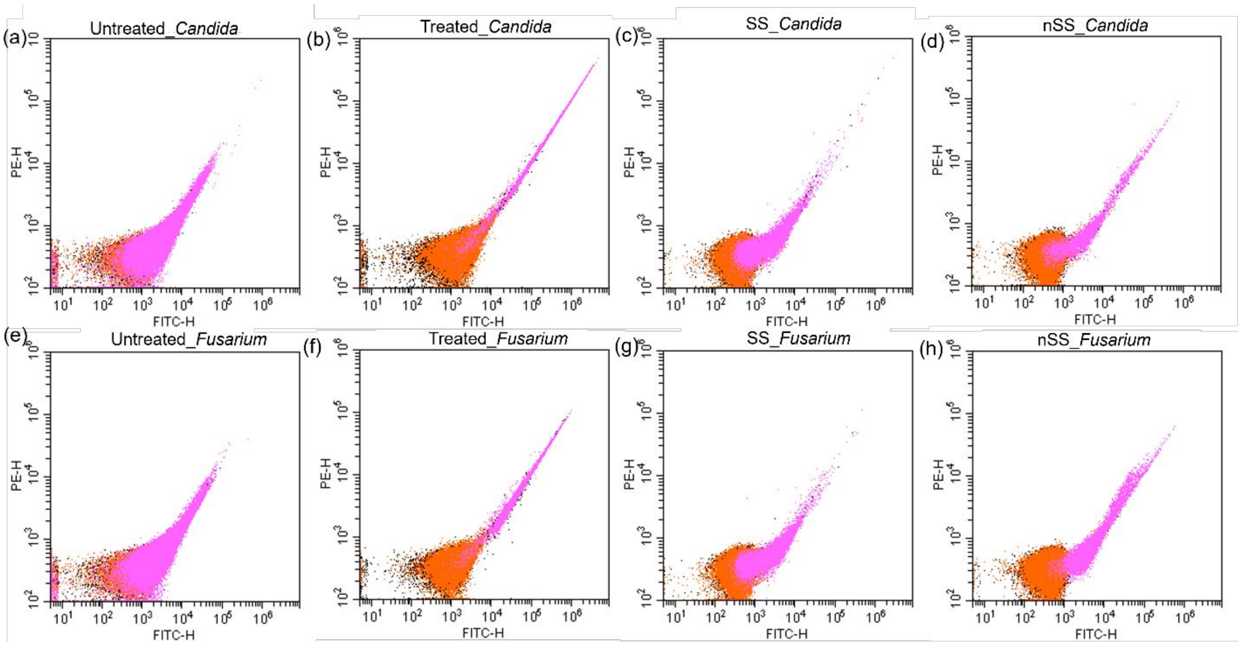

Figure S3: Membrane depolarization was assessed via flow cytometry (a-h). CCCP-treated cells (b, f) served as positive control samples. Green fluorescence (FITC) and red fluorescence (PE) of all cells are plotted on x and y axis, respectively. Gating on forward scatter (FSC) and granularity (side scatter, SSC) was used to identify non-budding *Candida* (c, d) or macroconidia *Fusarium* (g, h) in orange and budding Candida or microconidia Fusarium in pink.

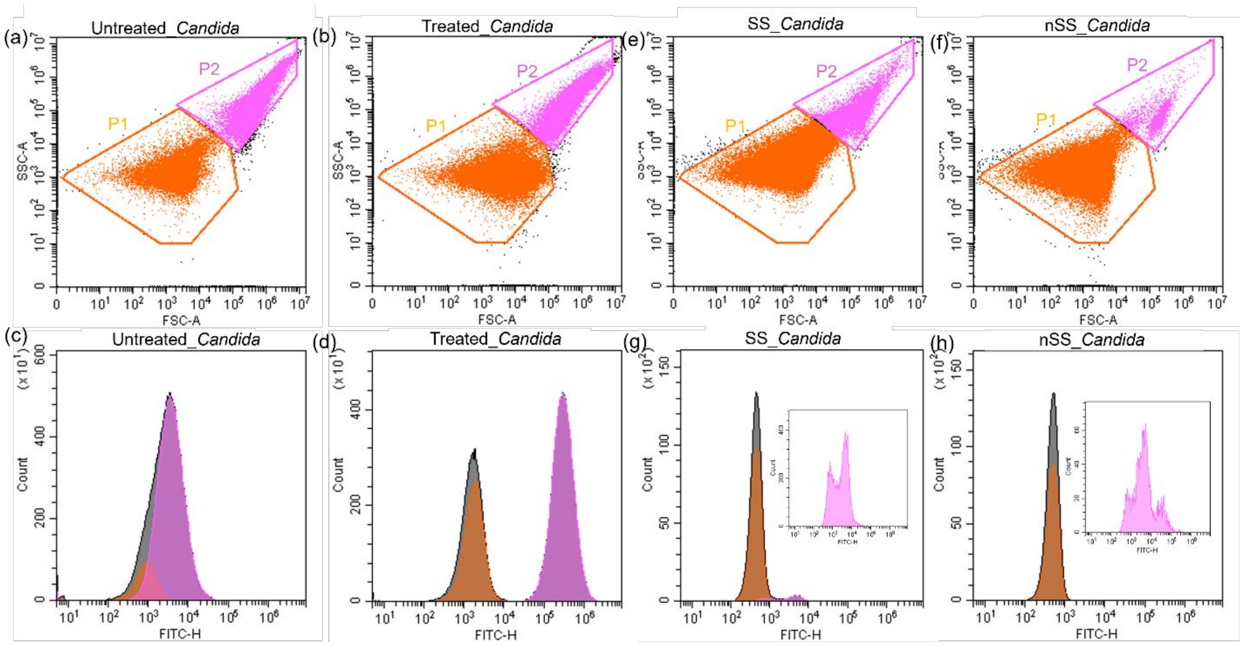

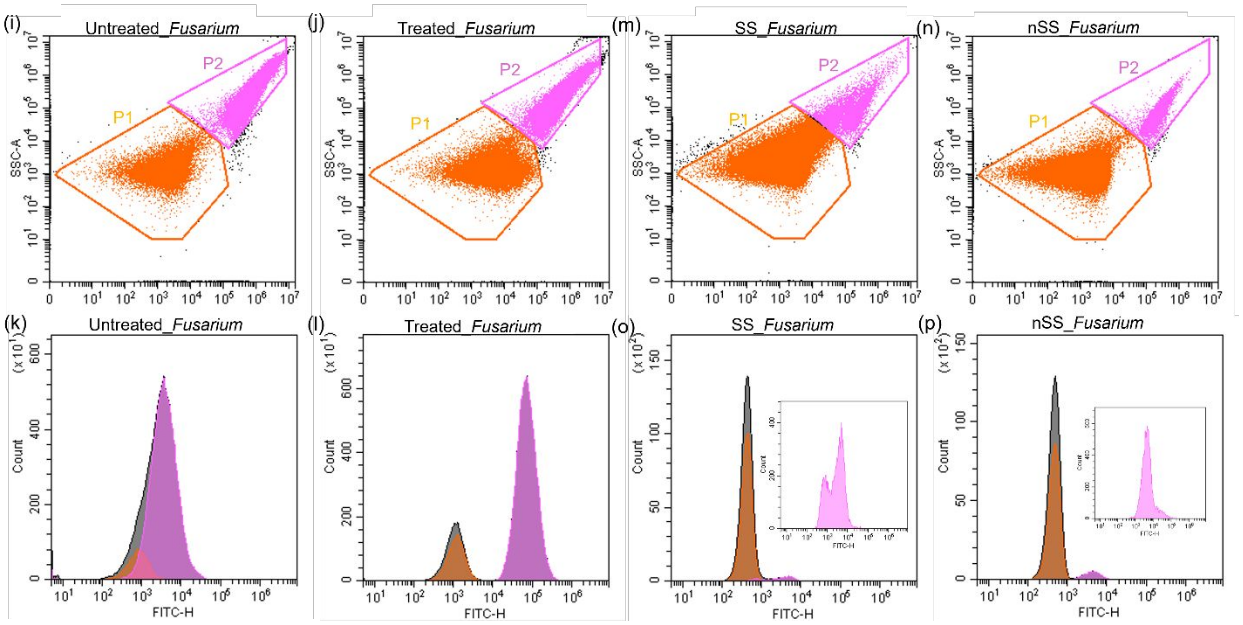

Figure S4: Representative raw data for ROS measurement using ROS-sensitive dye, DCFDA, in *Candida* (a-h) and *Fusarium* (i-p) Forward scatter (cell size) vs side scatter (cell granularity) plots for fungal cells. Gates (P1: non-budding/macroconidia orange, P2: budding/microconidia pink, ungated are gray) for two distinctive pollutions were drawn from negative control. Flow cytometer histograms for green fluorescence shift of fungal cells after incubating with SS and nSS surfaces. Insets are used to show P2 population fluorescence for SS and nSS incubation since those populations are very small. Cells positive for ROS show increased fluorescence. Untreated cells and peroxide treated cells were used as negative and positive control samples, respectively.

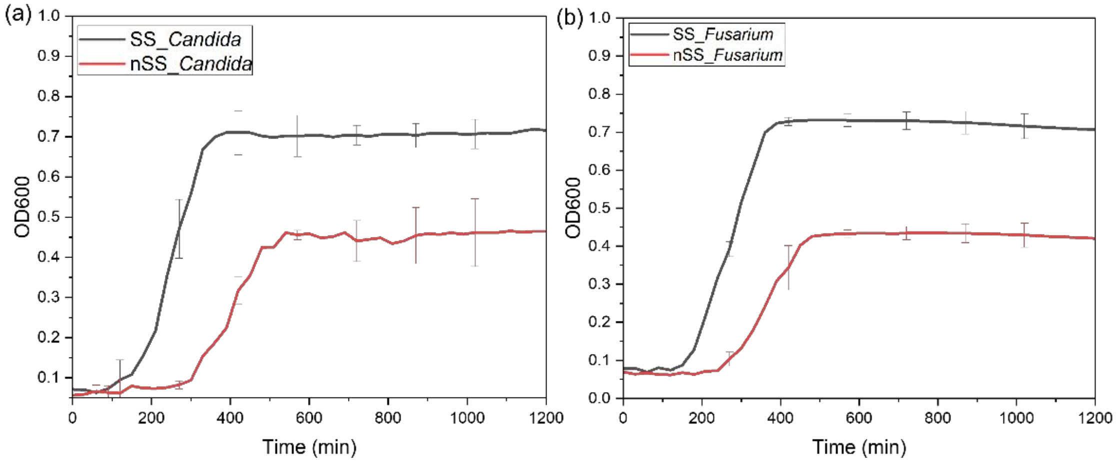

Figure S5: Fungal cell growth curves of *Candida albicans* (a) and *Fusarium oxysporum* (b) in dextrose media, measured in the presence of the control and nSS (n=3).

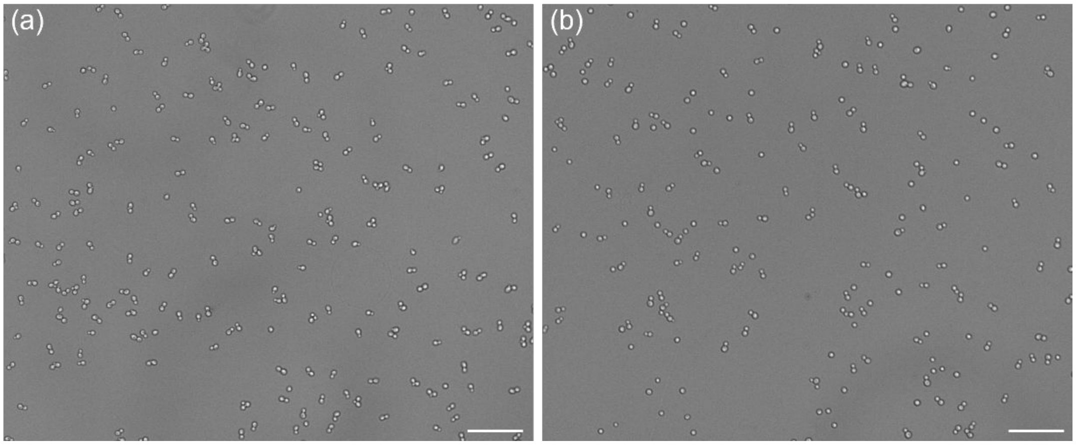

Figure S6: Optical images of untreated budding cells/microconidia of *Candida albicans* (a) and *Fusarium oxysporum* (b) after overnight culture at 30°C. Scale bar is 50 μm.
